# Cost and consequences of using 7.1 % chlorhexidine gel for newborn umbilical cord care in Kenya

**DOI:** 10.1186/s12913-021-06971-7

**Published:** 2021-11-19

**Authors:** Lecia Brown, Alan Martin, Christopher Were, Nandita Biswas, Alexander Liakos, Elena DeAngelis, Lee Alexandra Evitt

**Affiliations:** 1 Global Health, GSK, London, UK; 2 Present affiliation: Clinical Development, GSK, Pennsylvania Philadelphia, USA; 3 Value Evidence and Outcomes, GSK, Uxbridge, Middlesex UK; 4 Medical Department, GSK, Nairobi, Kenya; 5 Biostatistics, GSK, Pennsylvania Philadelphia, USA; 6 Present affiliation: Global Health Outcomes, ViiV Healthcare, Middlesex Brentford, UK

**Keywords:** Chlorhexidine, dry cord care, Kenya, omphalitis, cost-consequence

## Abstract

**Background:**

Omphalitis is an important contributor to neonatal mortality in Kenya. Chlorhexidine digluconate 7.1 % w/w (CHX; equivalent to 4 % w/w chlorhexidine) was identified as a life-saving commodity for newborn cord care by the United Nations and is included on World Health Organization and Kenyan Essential Medicines Lists. This pilot study assessed the potential resource savings and breakeven price of implementing CHX for neonatal umbilical cord care versus dry cord care (DCC) in Kenya.

**Methods:**

We employed a cost-consequence model in a Kenyan birth cohort. Firstly, the number of omphalitis cases and cases avoided by healthcare sector were estimated. Incidence rates and treatment effect inputs were calculated from a Cochrane meta-analysis of randomised clinical trials (RCTs) (base case) and 2 other RCTs. Economic outcomes associated with omphalitis cases avoided were determined, including direct, indirect and total cost of care associated with omphalitis, resource use (outpatient visits and bed days) and societal impact (caregiver workdays lost). Costs and other inputs were sourced from literature and supplemented by expert clinical opinion/informed inputs, making necessary assumptions.

**Results:**

The model estimated that, over 1 year, ~ 23,000 omphalitis cases per 500,000 births could be avoided through CHX application versus DCC, circumventing ~ 13,000 outpatient visits, ~ 43,000 bed days and preserving ~ 114,000 workdays. CHX was associated with annual direct cost savings of ~ 590,000 US dollars (USD) versus DCC (not including drug-acquisition cost), increasing to ~ 2.5 million USD after including indirect costs (productivity, notional salary loss). The most-influential model parameter was relative risk of omphalitis with CHX versus DCC. Breakeven analysis identified a budget-neutral price for CHX use of 1.18 USD/course when accounting for direct cost savings only, and 5.43 USD/course when including indirect cost savings. The estimated breakeven price was robust to parameter input changes. DCC does not necessarily represent standard of care in Kenya; other, potentially harmful, approaches may be used, meaning cost savings may be understated.

**Conclusions:**

Estimated healthcare cost savings and potential health benefits provide compelling evidence to implement CHX for umbilical cord care in Kenya. We encourage comprehensive data collection to make future models and estimates of impacts of upscaling CHX use more robust.

**Supplementary Information:**

The online version contains supplementary material available at 10.1186/s12913-021-06971-7.

## Background

Umbilical-cord infections (omphalitis) are an important contributor to neonatal mortality in low- and middle-income countries [1], where the newly cut umbilical cord can be an entry point for bacteria, causing newborn sepsis and death [2]. The causes of neonatal infection may be related to cord-care choices postpartum, including cultural practices of applying traditional substances, such as cow dung or ash, that may be harmful to the umbilical cord as part of postnatal care [3, 4]. Cord care also includes the use of gentian violet, silver sulphadiazine, topical antibiotics, and methylated and surgical spirits [3–5].

In 2012, the United Nations (UN) identified chlorhexidine digluconate 7.1 % w/w (CHX; equivalent to 4 % w/w chlorhexidine), an antiseptic agent with topical antibacterial activity, as a life-saving commodity for newborn cord care [6]. The UN called for pharmaceutical manufacturers to supply high-quality, affordable CHX that, if accessed more widely, could save 422,000 neonatal lives over 5 years [6]. In 2013, the World Health Organization (WHO) added CHX to its Model List of Essential Medicines for Children [7]. That year, the WHO also issued guidelines on umbilical cord care, based on evidence of efficacy and safety from three large community-based randomised clinical trials (RCTs) in low- and middle-income Asian countries [8–10]. These guidelines recommend daily application of CHX (during the first week of life) for cord care in home-birth settings with high neonatal mortality rates, and where it may replace potentially harmful traditional approaches to cord care [11]. In response to the UN call, and in line with the WHO guidelines, GlaxoSmithKline developed a CHX gel, in collaboration with Save the Children [12]. This was produced in single-use sachets. As part of the collaboration, a managed access programme investigated the use of CHX gel for newborn umbilical cord care across 21 healthcare facilities in Bungoma County, Kenya. Feedback from healthcare providers and mothers indicated positive outcomes in terms of reduced newborn infections and ease of use [13]. There is locally manufactured multi-application CHX available, supplied in a bottle or tube.

In higher-income countries, keeping the cord clean and dry – ‘dry cord care’ (DCC) is sufficient under most circumstances [14]. CHX is associated with reduced infection and omphalitis rates compared with DCC in studies conducted in low-resource settings [15–17]; therefore, widespread use could lead to reductions in subsequent healthcare costs. However, DCC is not necessarily the standard of care in low- and middle-income countries, where mothers may frequently use a potentially harmful product on the cord [3–5]. Despite recommendations for use from international organisations, there is limited analysis on the financial implications of the introduction and national scale-up of CHX use for neonatal cord care. Generation of such data could help inform financial decision-making.

However, there are concerns regarding the safety and effectiveness of CHX. The WHO issued a drug safety alert in 2019 following at least 40 recorded incidents, across nine sub-Saharan countries, of CHX misuse in the eye when mistaken for eye drops and ointments, leading to injury and, in some cases, blindness [18]. Additionally, data from two clinical trials in African countries were more equivocal in terms of impact on mortality of CHX versus DCC, than the previously mentioned Asian studies [16, 17].

Kenya, located in East Africa, has a higher neonatal mortality rate than the average globally and of low- to middle-income countries, at 20 deaths per 1000 live births [19]. Neonatal care is predominantly provided by the public sector, but also through private and faith-based organisations (FBO) [20]. In 2014, the Kenyan Ministry of Health began to develop national guidelines on newborn cord care and the use of CHX. This led to the inclusion of CHX in the Kenyan Essential Medicines List [21]. Given the WHO recommendations supporting the use of CHX in home-birth settings with high neonatal mortality rates and Kenyan national clinical guidelines, understanding the economic consequences of upscaling the use of CHX may be helpful in supporting investment decisions. As with most preventative interventions, not all patients will derive benefit at an individual level; however, at a population level there will be a proportion of patients who will benefit due to avoidance of infections. These analyses are viewed as relevant for Kenya because there is variation in neonatal mortality rates between counties [22], and despite the move towards scale-up of CHX as part of routine postnatal care, a prominent proportion of neonates still receive improper cord-care hygiene [4]. Evidence suggests that supply of locally manufactured CHX is still fragmented and barriers to widespread use persist [13].

The goal of this pilot study was to provide preliminary evidence of the financial and clinical implications associated with the use of CHX, through a cost-consequence analysis assessing the implementation of the gel for neonatal umbilical cord care in Kenya. It is hoped that this will be used as the foundation for future studies that will utilise more extensive datasets and additional sources to reduce any uncertainty surrounding the model outcomes. Ultimately, such data could be used to inform healthcare systems during decision-making regarding the treatment of omphalitis.

## Methods

### Overview

This cost-consequence analysis assessed clinical and economic outcomes for CHX gel compared with DCC for neonatal umbilical cord care in Kenya, from both healthcare system and societal perspectives. Clinical outcomes were number of cases and number of cases avoided by sector. Economic outcomes were direct, indirect and total cost of care for omphalitis, resource use (number of bed days and outpatient visits) and societal impact (workdays lost). Breakeven analysis was performed to ascertain the price of CHX that would lead to a net-neutral budget outcome.

### Data collection and analysis

#### Model structure

**Fig. 1:**
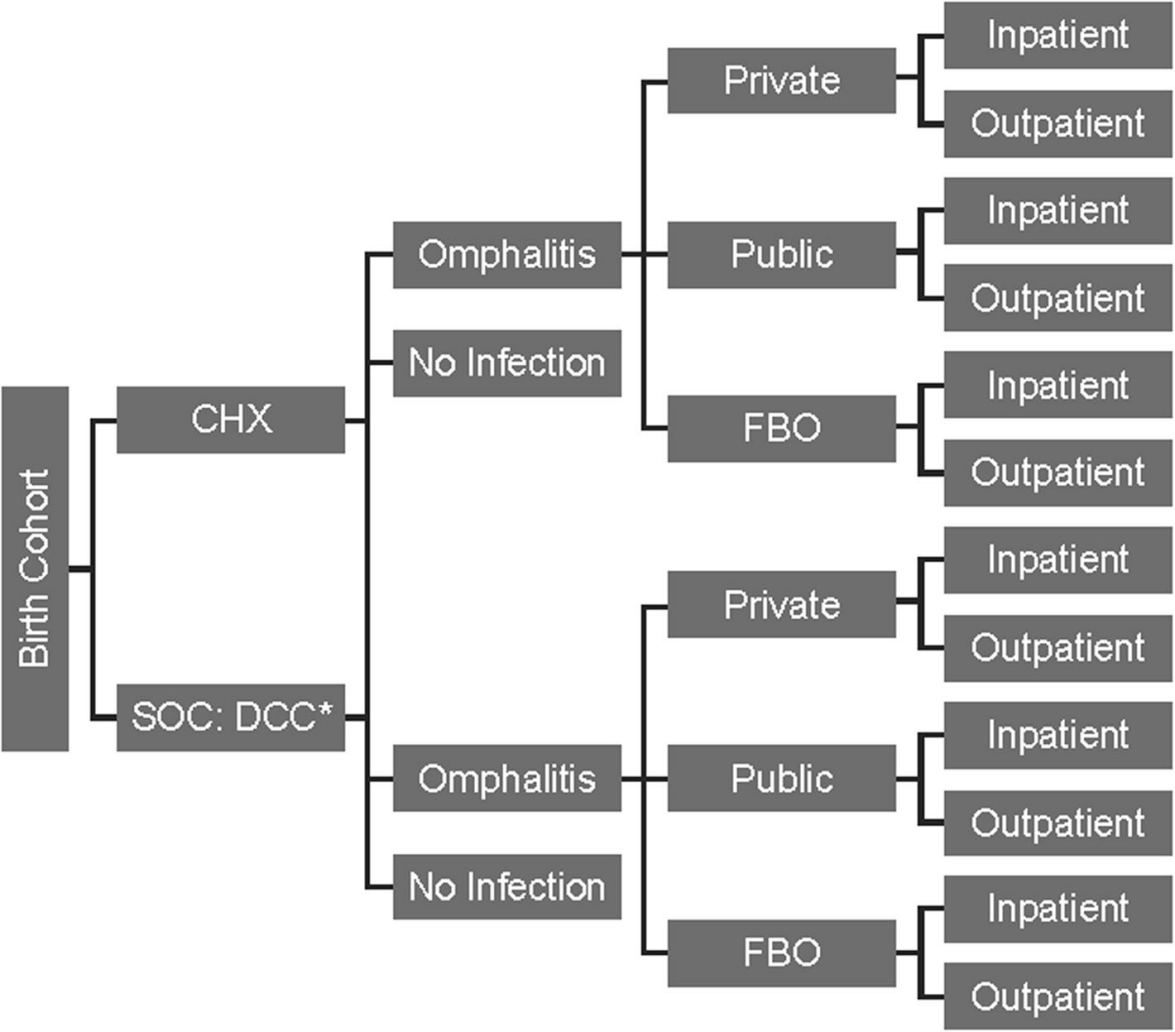
Cost-consequence model design. *Although DCC represented SOC in this model, it is not viewed as being realistically representative of the SOC in Kenya, where harmful substances such as methylated spirits, ash or saliva, are often used [3–5]. CHX, chlorhexidine; DCC, dry cord care; FBO, faith-based organisation; SOC, standard of care

The model is a de novo spreadsheet calculation (Microsoft Excel) analysing the frequency of omphalitis cases and financial implications (cost-consequence) associated with implementing either DCC or CHX as cord care strategies in neonates (Fig. 1). For each strategy, the number of cases of omphalitis is calculated, followed by the overall cost of the infection accounting for the sector providing omphalitis treatment (private, public, FBO) and the setting of care (inpatient or outpatient).

#### Model inputs and data analysis

Key model inputs are summarised below; further details are provided in Supplementary Tables 1, 2, 3, 4, 5,6.

Patient population: Birth cohort of 500,000 in Kenya for 1 year, with an average birth weight of 3.3 kg [23]. The population size was selected as a sample to provide an indication of the impact of the intervention; the aim was not to represent the entire annual birth population in Kenya.

Clinical efficacy: The inputs used to determine the clinical efficacy of DCC and CHX for the base case analysis were taken from a Cochrane systematic literature review based on three RCTs conducted in Asian countries [8–10, 24]. The Cochrane review provides the largest pool and most robust data for clinical efficacy and was used by the WHO to support its recommendation for use of CHX versus DCC [11, 24]. Additionally, two RCTs, conducted in African countries, were used to determine the most relevant and applicable efficacy inputs to be used [16, 17]. Further justification for the use of this literature is shown in Supplementary Table 7. Relative risk of omphalitis - due to limitations on data reporting within publications, an overall relative risk for omphalitis for CHX versus DCC could not be calculated, and for the base case was assumed to be equivalent to the relative risk reported in the Cochrane review for the “redness extending to the skin” categorisation (RR:0.73). This is a conservative effect size when compared with that reported for other severity categories [24]. Omphalitis case inputs - the inputs to determine the number of cases of omphalitis calculated in the model were taken from the efficacy of DCC and CHX from the Cochrane review comparing the two cord care approaches [24] (Supplementary Table 1). As an overall incidence rate of omphalitis was not reported in the Cochrane review, a weighted average was calculated of incidence rates of all severities of omphalitis associated with the DCC intervention arm from the three randomised control trials included in the Cochrane review [24].

Cord care treatment costs: The aim of the present analysis was to determine the breakeven budget-neutral price of CHX, therefore, the acquisition cost has not been included. As there is a generic local manufacturer in Kenya, it was assumed that all CHX was locally manufactured. For locally manufactured CHX, which is delivered in a multiple-application formulation, the number of days of treatment in each tube becoming wastage is also assumed. DCC was assumed to be associated with a zero cost. Cost inputs were converted from Kenyan shillings (KSH) to USD at an exchange rate of 100.83 KSH per 1 USD (Statistics, 2018) (Supplementary Table 2).

Proportion of patients per sector: No data could be identified in the literature; therefore, assumptions based on clinical opinion were used for the proportion of omphalitis cases treated in different sectors (Supplementary Table 1).

Inpatient and outpatient resource use and costs: The proportion of patients treated in each setting was based on clinical opinion. Costs for inpatient days and outpatient visits were populated drawing on the costs for Kenya provided in WHO-CHOICE 2008 [25], inflated to 2019 cost using the annual Kenya Consumer Price Index [26]. Costs accounted for personnel, capital and (for inpatient care) food [25, 26]. To reflect the structure of Kenya’s outpatient facilities, health centres (no beds), health centres (with beds) and primary level hospitals were averaged to provide an input for primary level care costs. Outpatient care was averaged across primary level, secondary level and teaching hospitals, weighted by the proportion of patients treated in each setting (Supplementary Table 2).

Medication costs: These model inputs were based on WHO guidelines, Kenya Paediatric guidelines and expert clinical opinion and assume the medication used and duration of treatment for omphalitis is influenced by sector and setting of care [4, 27] (Supplementary Tables 2 and 3). Costs were calculated using the cost of the medication by pack/vial, dosage prescribed and treatment length (in days).

Non-medication costs: Laboratory test costs associated with treating omphalitis were sourced from the literature [28], with FBO sector laboratory costs assumed to be one-third of private sector costs. To reflect the reality of the clinical environment, other non-medication costs were also included based on clinical opinion. This included the number and cost of consumables, and number of laboratory tests associated with treating omphalitis, both assumed to be equivalent across sectors (Supplementary Tables 4 and 5).

Societal factors and productivity: To understand the caregiver/family impact of caring for a child with omphalitis in Kenya, the model also estimated the impact on productivity and private financial losses based on the average gross salary and workdays lost through caring for a neonate with omphalitis. (Supplementary Table 6).

#### Model assumptions

Assumptions relating to incidence rates of omphalitis with DCC, comparative efficacy of CHX versus DCC, medication use and productivity loss are listed in Table 1. Further details on model assumptions are included in the Supplementary Tables.

**Table 1:** Key assumptions made in the CHX cost-consequence model

Input	Assumptions
Incidence rates of omphalitis with DCC	DCC, and the associated incidence rate of omphalitis, reflects the standard of care and therefore the rate in clinical practice in Kenya. This is due to the limitation in data available from robust clinical trials limiting the comparison of CHX to DCC only. Absolute cases of omphalitis avoided may be underestimated compared with clinical practice.
Comparative efficacy: relative risk of omphalitis	The relative risk of omphalitis for CHX versus DCC in the base case is assumed based on data from the Cochrane review for the least severe category of omphalitis.
Comparative efficacy: mortality	Not included in the model. Therefore, the effect of CHX is only applied to rates of omphalitis.
Medications to treat omphalitis	Assumed to predominantly follow clinical opinion. Clinical practice may differ from guideline recommendations.
Productivity loss	Calculated based on the average salary in Kenya and therefore average cost of time lost.

*CHX* chlorhexidine treatment; *DCC* dry cord care

#### Analysis perspective

For the primary analysis a healthcare system perspective was taken to estimate the costs and benefits of implementing CHX for the Ministry of Health in Kenya or relevant healthcare provider, both overall and by sector (private, public or FBO) and setting of care (inpatient or outpatient). This included medication cost and costs associated with healthcare resource use in the treatment of omphalitis. In addition, a scenario with a societal perspective on the introduction of CHX was also analysed. This included the impact on productivity and workdays lost associated with time off work due to the consequences of different cord care interventions. The time horizon was 1 year, so no discounting was applied. Input values and associated sources for the base case (direct costs only) and scenario (which also included indirect costs) are described above and in Supplementary Tables 1, 2, 3, 4, 5, 6.

The analysis aimed to determine the impact on the costs of omphalitis treatment. Therefore, the cost of CHX was not included. Instead, cost of CHX was addressed as part of the breakeven analysis described below.

The analysis compared two treatment approaches: DCC (assumed to be standard of care) and CHX application (Fig. 1). Patients were assumed to receive either CHX or DCC; however, in many low- and middle-income countries, mothers often choose to apply other remedies; such as methylated spirits and ash on the cord [3, 4]. As this analysis used data that reflects the WHO guidelines on recommended cord care [29] and the available clinical trial data [16, 17, 24], it does not include the use of other substances; therefore, it represents a conservative analysis. Neonatal mortality was not included in the model, only the effect of CHX on rates of omphalitis.

#### Breakeven analysis

Breakeven analysis was performed to ascertain the price of CHX that would lead to a net-neutral budget outcome, where any CHX price below this would allow savings from reducing omphalitis cases to be realised. This analysis was conducted from both perspectives, considering direct cost savings only and both direct and indirect cost savings.

#### One-way sensitivity analysis

A one-way sensitivity analysis was programmed to evaluate the robustness and sensitivity of results to changes in parameter values, to identify those parameters with the greatest influence on model outcomes. Upper and lower parameter values were based on ranges informed by plausible variation (± 20 %) and are shown in Supplementary Tables 1, 2, 3, 4, 5, 6. 

## Results

### Clinical outcomes

#### Cases of omphalitis by sector

**Fig. 2:**
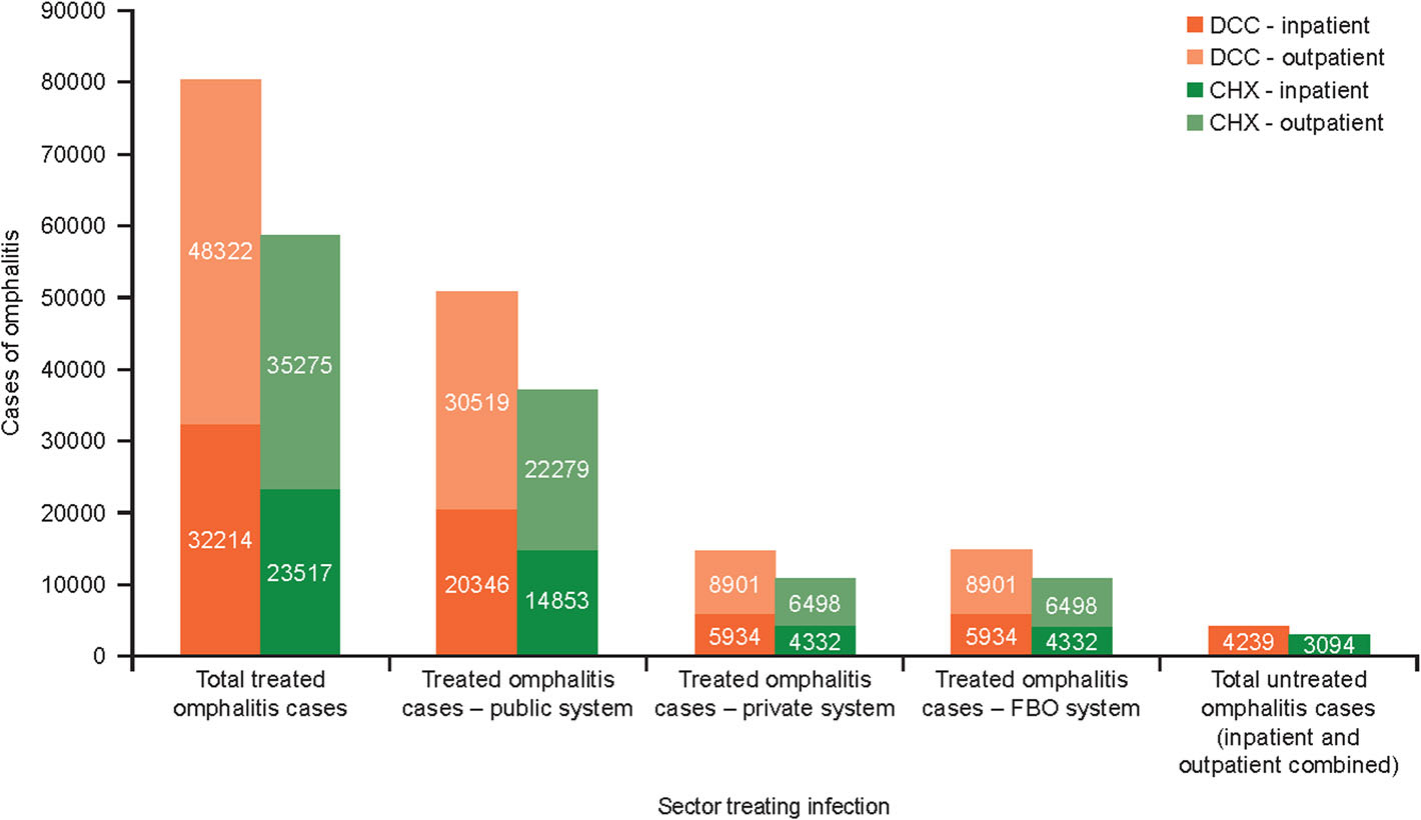
Model-predicted cases of omphalitis by sector treating the omphalitis infection for a birth cohort of 500,000 for 1 year. *Abbreviations:* *CHX* chlorhexidine, *DCC* dry cord care, *FBO* faith-based organisation

The model estimated that in a birth cohort of 500,000 newborns in Kenya, with DCC as routine cord care for newborns, approximately 85,000 omphalitis cases would occur, with 51,000 treated in the public sector, 15,000 in both the private and FBO systems, and 4000 untreated. Of the treated cases, 48,000 cases would be seen as outpatients and 32,000 as inpatients. With CHX, approximately 62,000 omphalitis cases would occur, with 37,000 treated in the public sector, 11,000 in both the private and FBO systems and 3000 untreated. Of the treated cases, 35,000 cases would be seen as outpatients and 24,000 as inpatients (Fig. 2).

#### Cases of omphalitis avoided

The number of omphalitis cases avoided in a population of 500,000 newborns in Kenya was estimated based on base case inputs shown in Supplementary Tables 1, 2, 3, 4, 5, 6. Some of the inputs included were: the omphalitis incidence rate with DCC (0.170); CHX efficacy (relative risk vs. DCC; 0.730); and the proportion of patients with omphalitis treated in each system (public system, 0.60; private system, 0.175; FBO, 0.175; untreated, 0.05). Based on these inputs, the cost-consequence model estimated that CHX introduction to a birth cohort of 500,000 newborns in Kenya may lead to the avoidance of approximately 23,000 omphalitis cases compared with DCC (Fig. 2). The scale of these reductions was consistent across the three treatment sectors, with CHX reducing incidence of omphalitis by approximately 14,000 cases in the public setting and 4000 cases in the private and FBO settings. Across treatment sectors, the model estimated that the use of CHX over DCC can avoid approximately 9000 omphalitis cases in the inpatient setting and approximately 13,000 cases of omphalitis in the outpatient setting.

### Economic outcomes

#### Costs

Due to the modelling assumptions, DCC was associated with zero acquisition costs. Acquisition cost of CHX was not included in the analysis of direct costs (i.e. monetised healthcare resource use only, as the analysis looked to estimate the breakeven cost for CHX). CHX usage led to a decreased cost of care for omphalitis of approximately 590,000 USD compared with DCC (Table 2). Medication, hospitalisation, outpatient and non-medication costs (predicted in USD) associated with omphalitis (in a birth cohort of 500,000) were all lower with the use of CHX compared with DCC. The most significant subcategory of direct cost savings was hospitalisations, which contributed 399,585 USD of savings. The savings for medication costs were 39,723 USD, outpatient costs were 29,238 USD, and non-medication costs were 119,997 USD (Table 2). When including indirect costs associated with salary loss, the estimated total cost savings increased to over 2.5 million USD when using CHX over DCC. The most significant subcategory of indirect cost savings was salary loss associated with having a child with omphalitis; the costs associated with this were predicted to be 7,879,007 USD for DCC and 5,751,675 USD for CHX treatment, corresponding to a saving of 2,127,332 USD.

**Table 2:** Model-predicted direct and indirect cost summary for CHX versus DCC for a birth cohort of 500,000 for 1 year

	DCC (USD)	CHX (USD)	Difference^a^
Omphalitis treatment			
Medication cost	147,121	107,398	-39,723
Hospitalisation costs	1,479,943	1,080,358	-399,585
Outpatient costs	108,289	79,051	-29,238
Non-medication costs	444,433	324,436	-119,997
TOTAL direct costs	2,179,787	1,591,244	-588,542
Salary loss due to child with omphalitis	7,879,007	5,751,675	-2,127,332
Total direct and indirect costs	10,058,793	7,342,919	-2,715,874

*CHX* chlorhexidine; *DCC* dry cord care; *USD* US dollar

^a^Negative value indicates a cost saving associated with CHX compared with DCC

#### Breakeven analysis

**Fig. 3:**
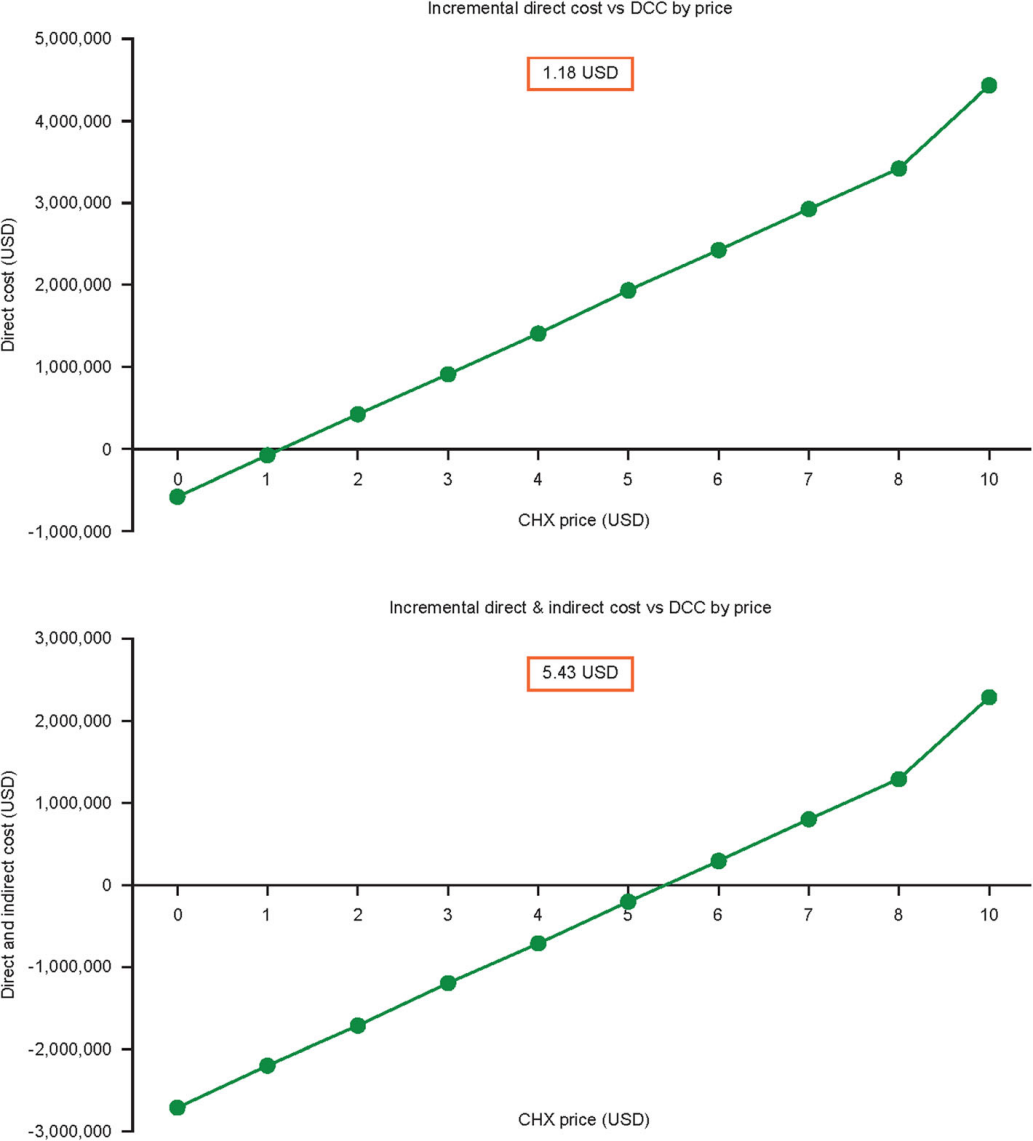
Breakeven analysis of direct cost and a combination of direct and indirect costs of CHX compared with DCC. *Abbreviations:* *CHX* chlorhexidine, *DCC* dry cord care, *USD* US dolla

Taken from the healthcare system perspective, when considering direct costs only, the estimated breakeven price of CHX when used in place of DCC was 1.18 USD per course. Therefore, use of CHX at any price below this could lead to direct cost savings to the healthcare system. When considering both direct and indirect costs (from a societal perspective) the breakeven price of CHX became 5.43 USD per course (Fig. 3**)**. The acquisition cost of CHX was not included in this analysis.

#### Resource use and societal impact

It was estimated that DCC and CHX, respectively, led to 48,322 and 35,275 outpatient visits; 161,072 and 117,583 bed days; and 423,874 and 309,428 workdays lost. Therefore, in terms of resource use, approximately 13,000 outpatient visits could be avoided and approximately 43,000 bed days saved through use of CHX over DCC. When assessing the societal impact, approximately 114,000 fewer workdays were lost due to caring for a neonate with omphalitis for CHX versus DCC (Table 3).

**Table 3:** Model-predicted omphalitis-related resource use associated with CHX and DCC for a birth cohort of 500,000 for 1 year

	DCC	CHX	Difference
**Workdays lost**	423,874	309,428	-114,446
**Bed days**	161,072	117,583	-43,489
**Outpatient visits**	48,322	35,275	-13,047

*CHX* chlorhexidine; *DCC* dry cord care

### Impact of changing parameter values on cost of CHX versus DCC

**Fig. 4:**
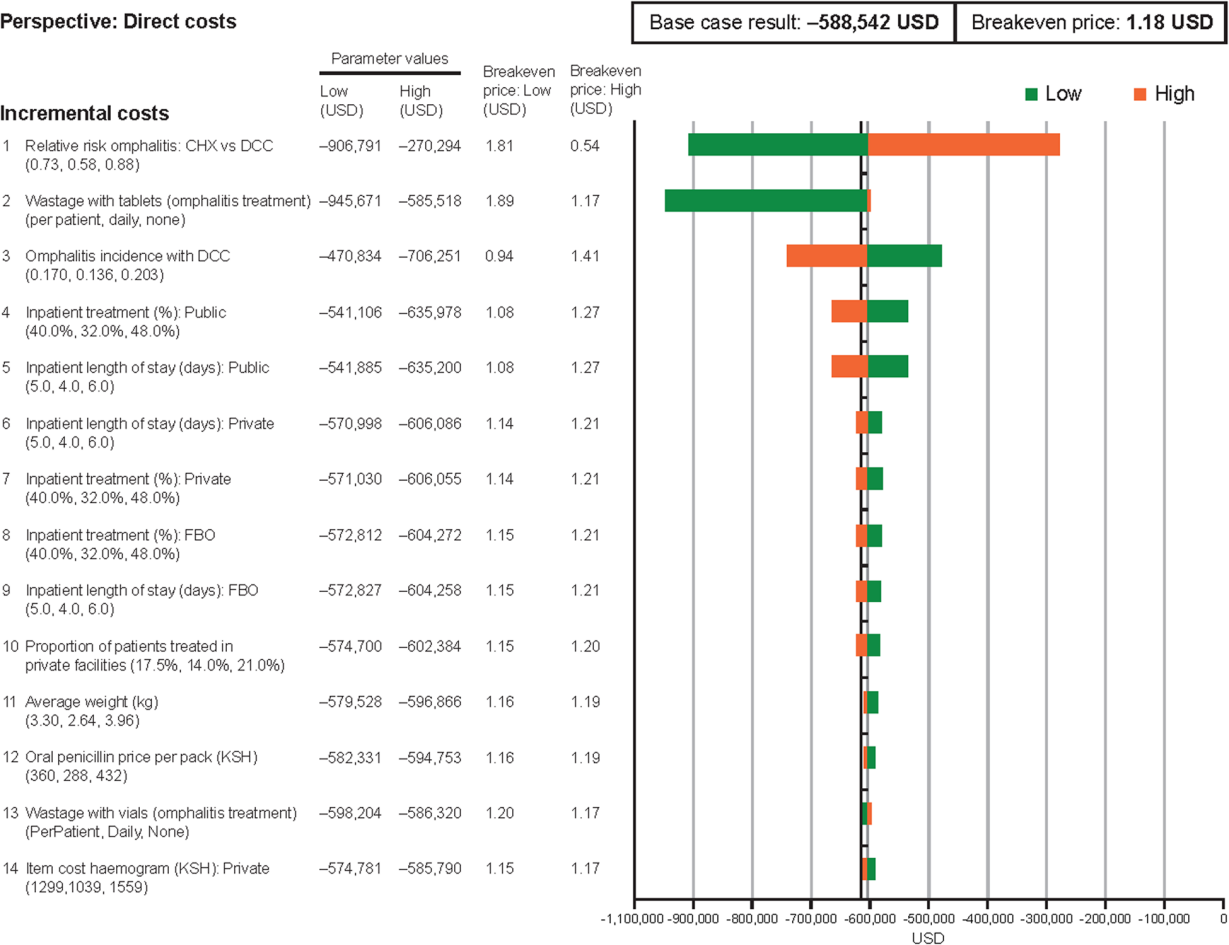
One-way sensitivity analysis of the impact that changes in direct costs will have on the economic evaluation output. *Abbreviations:* *CHX* chlorhexidine treatment, *DCC* dry cord care, *FBO* faith-based organisation, *KSH* Kenyan shilling, *USD* US dollar

**Fig. 5:**
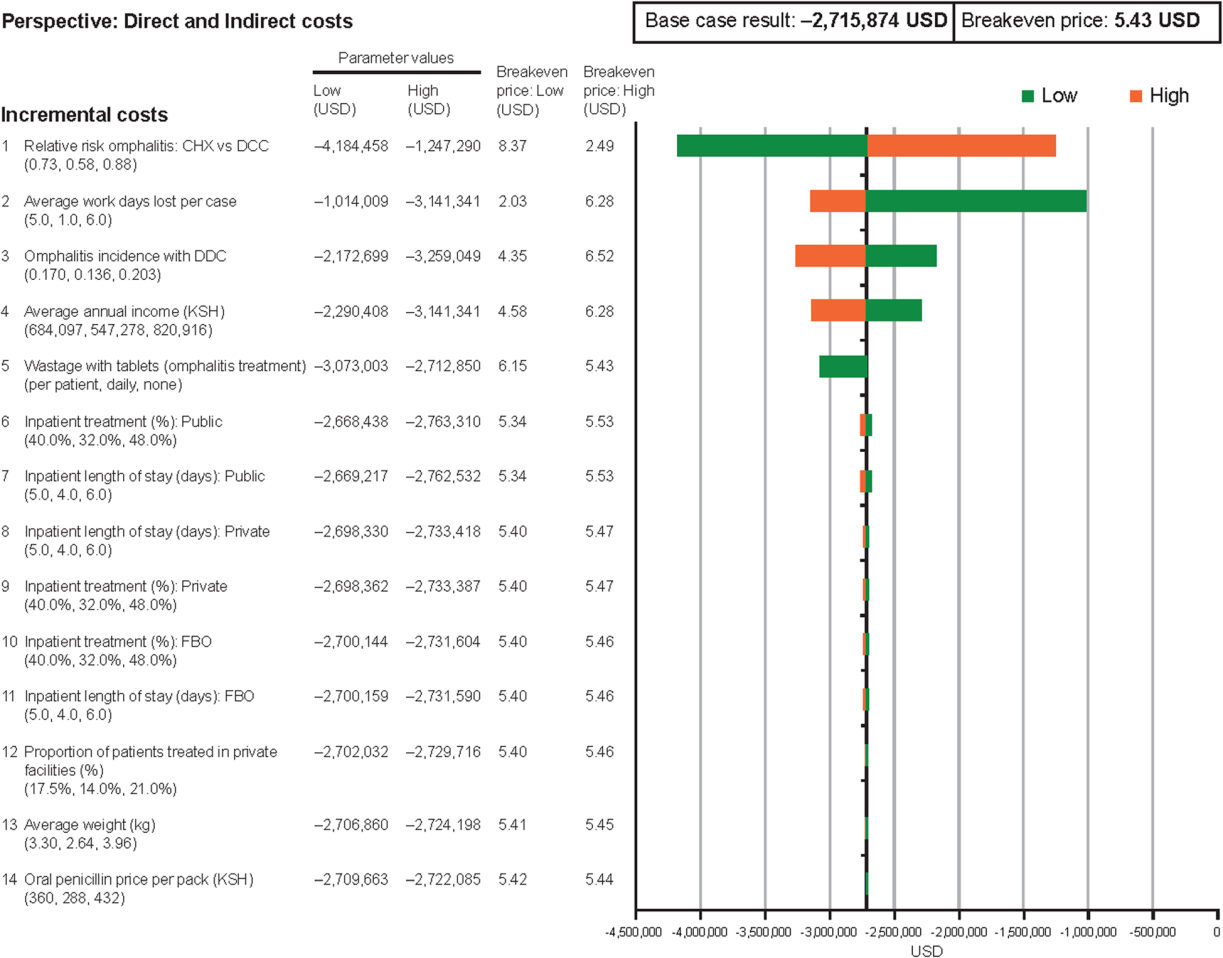
One-way sensitivity analysis of the impact that changes in direct and indirect costs will have on the economic evaluation output. *Abbreviations:* *CHX* chlorhexidine treatment, *DCC* dry cord care, *FBO* faith-based organisation, *KSH* Kenyan shilling, *USD* US dollar

One-way sensitivity analysis was programmed to evaluate the robustness and sensitivity of results to changes in parameter values, to identify those parameters with the greatest influence on model outcomes. Based on this, the 14 most influential parameters are presented in Fig. 4, when assessing direct costs only, and Fig. 5, when assessing both direct and indirect costs. The acquisition cost of CHX was not included in this analysis. Overall, the reduction in total healthcare spend on omphalitis treatment with CHX versus DCC is robust to changes in parameter values, remaining close to a 590,000 USD reduction in the base case for most parameters for direct costs; the breakeven price remains close to the base case of approximately 1.18 USD, following changes in most parameters (Fig. 4). The most influential parameter was the relative risk of omphalitis with CHX versus DCC for both direct costs only and also if indirect costs are included. There are still cost savings even after the relative efficacy was reduced by 20 % (relative risk changed from 0.73 to 0.88), when considering direct costs only (lower and upper bounds for cost saving: 906,791–270,294 USD) (Fig. 4) and also if indirect costs are included (4,184,458–1,247,290 USD) (Fig. 5). Other influential parameters for direct costs included wastage of antibiotic tablets (penicillin) for omphalitis treatment (945,671–585,518 USD); and omphalitis incidence with DCC (470,834–706,251 USD). When indirect costs are included, average workdays lost per case (1,014,009–3,141,341 USD) and average annual income (2,290,408–3,141,341 USD) also become influential, since these determine the extent of the societal cost due to time off work from omphalitis cases.

## Discussion

CHX, identified as a life-saving commodity by the UN, is included in the Kenya Essential Medicines List and recommended for neonatal care in the guidelines [21, 27, 29]. Despite recommendations by the Kenyan Ministry of Health, barriers to widespread use of CHX still exist in Kenya, with many neonates not receiving appropriate cord care [4, 13]. Considering the prevalence and burden of omphalitis, the current pilot study was conducted as an initial assessment of the financial implications associated with CHX implementation for cord care in a birth cohort in Kenya, and to serve as a foundation for future studies that will utilise more extensive datasets and additional sources to reduce uncertainty surrounding the model outcomes.

Using the best available evidence from published literature and expert clinical opinion, the results of this model suggest that the use of CHX versus DCC can lead to a reduced number of omphalitis cases in neonates and therefore provides cost savings when considering the cost of cord care and omphalitis treatment [8–10, 24]. The data from this pilot study, provides evidence that introducing CHX use in a birth cohort of 500,000 newborns in Kenya could lead to the avoidance of nearly 23,000 omphalitis cases compared with DCC, with reductions consistent across treatment sectors. Omphalitis is a significant contributor to neonatal mortality [1]; although neonatal mortality was not included in the model, this reduction in omphalitis cases with CHX use is expected to lead to improved clinical outcomes in neonatal cord care and ultimately, a reduction in neonatal deaths. This is substantiated by findings from the UN that showed that widely available and affordable CHX could save 422,000 neonatal lives over 5 years [6]. Additionally, trials conducted in other low- to middle-income countries showed that CHX reduced the risk of omphalitis compared with DCC, highlighting the potential benefit of CHX implementation [16, 17].

Despite the potential life-saving benefits of widespread implementation of CHX for cord care, minimal analysis investigating the financial implications of CHX use has been conducted; our model provides a preliminary cost-consequence analysis of CHX implementation in a low- to middle-income country. Findings suggest substantial cost-savings to healthcare systems with implementation of CHX as standard of care for neonatal cord care instead of DCC. We estimated that up to 590,000 USD could be saved following CHX implementation, when compared with DCC and considering direct costs. In fact, the cost savings associated with CHX use versus DCC may be understated as the birth cohort of 500,000 used in this analysis is approximately 2.6-fold lower than the projected births in Kenya, based on data for 2014 [30]. As hospitalisations were identified as the most substantial subcategory of direct cost savings, implementation of CHX treatment could lead to extensive future savings in government healthcare facilities, which have been identified as important distribution points for CHX [31]. After accounting for the savings associated with the healthcare system’s direct resource use, the cost of CHX at which its use becomes budget neutral (i.e. off-setting the savings through resource use reduction) was 1.18 USD; CHX use instead of DCC at a price up to this threshold was found to lead to a direct cost saving to the healthcare system (without accounting for costs associated with purchasing other cord care products, such as methylated spirits). These data provide supporting evidence of the value that CHX could bring to the healthcare system for healthcare providers and manufacturers, which can be used to guide fair pricing regulation and policy [32]. Of note, the aforementioned budget neutrality does not take into account the potential social and health-related quality of life benefits that would likely come from reducing rates of omphalitis and improving neonatal care, and this should also be factored into healthcare decision-making. One-way sensitivity analysis findings suggested that overall, the monetised resource use reduction in total healthcare spend in Kenya with CHX versus DCC is robust to changes in a wide range of parameters and therefore, so is the breakeven price of CHX.

As this study represents a preliminary analysis, there are some limitations to consider. First, there was a paucity of data available for many of the model inputs, so assumptions and expert clinical opinion were used. These include assumptions on CHX treatment duration, that it would be properly administered, and its real-world effectiveness. However, the impact of this may not be as large as expected; as described above, the cost saving with CHX was robust to changes in the parameters based on assumptions guided by clinical opinion. Second, despite DCC representing the standard of care in this model, it is not viewed as being realistically representative of the standard of care in Kenya, where substances such as gentian violet, silver sulphadiazine, topical antibiotics and surgical methylated spirits, or even potentially more harmful substances such as ash or saliva, are often used [3–5]. Therefore, the model likely provides a conservative estimate of resource use and cost savings, as CHX gel was compared with DCC rather than the application of these other substances. This may mean that the cost savings were underestimated in this model. Third, indirect costs may have been overestimated in this model due to assumptions about productivity loss and standardised salary. Kenya has many workers who are paid daily rather than receiving a salary, and similarly, a proportion of parents may not be employed or may have chosen to take planned absence from work to care for their newborn, irrespective of whether they have omphalitis or not. All of these may contribute to an overestimation of the impact on productivity and, therefore, this model provides a notional estimate of indirect costs.

Healthcare system decision-makers need also consider some wider issues around the implementation of CHX. In relation to safety, both liquid and gel formulations have been associated with eye injuries when mistaken for eye drops and ointments, leading the WHO to warn all those involved in distributing and administering CHX to take appropriate steps to ensure its correct use [18]. National budget allocation for CHX implementation must also be provided for, as part of the UN recommendation/call to action [6].

It is hoped that the potential cost savings highlighted in this pilot study will encourage more comprehensive data generation to address the current information gaps around CHX effectiveness, epidemiological data and patterns of omphalitis care, thus making future models more robust and inform health systems further. To obtain a more holistic picture of the health impact of different cord care strategies it would be of interest to include humanistic factors such as health-related quality of life; and the effect of the stress and anxiety caused by omphalitis on parents and carers. Such data may encourage healthcare systems to overcome existing barriers to widespread implementation of CHX [13].

## Conclusions

These preliminary findings, based on the best available evidence, indicate that use of one course of CHX priced up to the breakeven price of 1.18 USD for direct costs only and 5.43 USD when including indirect costs could lead to cost savings compared with DCC, while also improving clinical outcomes in neonatal cord care. Reduction in healthcare costs and the breakeven price were robust to input parameter changes. Due to the conservative nature of the model, cost savings, and therefore the budget-neutral price, may have been underestimated and further analyses are encouraged. Nevertheless, this study strongly supports the need for further studies examining the use of CHX in developing countries.

## Supplementary Information

**Additional file 1: Supplementary Table S1**. Model inputs for DCC clinical efficacy, CHX treatment assumptions and sector care for patients contracting omphalitis.

**Additional file 2: Supplementary Table S2**. Model inputs for hospitalisation (inpatient) and outpatient resource costs and medications costs (KSH).

**Additional file 3: Supplementary Table S3**. Model inputs for proportion of patients treated with each drug regimen.

**Additional file 4: Supplementary Table S4**. Model inputs for non-medication costs.

**Additional file 5: Supplementary Table S5**. Model inputs for number of laboratory tests and consumable non-medication items conducted per case of omphalitis.

**Additional file 6: Supplementary Table S6**: Model inputs for indirect costs.

**Additional file 7: Supplementary Table S7**: Rationale for the included literature in this cost-consequence analysis.

## Data Availability

The datasets used and/or analysed during the current study are available from the corresponding author on reasonable request.

## Abbreviations

CHX: Chlorhexidine
DCC: Dry cord care
FBO: Faith-based organisation
KSH: Kenya shillings
UN: United Nations
USD: US dollar
WHO: World health Organization
