# Supplementary material for: Cost and consequences of using 7.1 % chlorhexidine gel for newborn umbilical cord care in Kenya
Source: BMC Health Serv Res. 2021 Nov 19;21:1249. doi: 10.1186/s12913-021-06971-7 (PMC8603569; doi:10.1186/s12913-021-06971-7)
Supplement: Supplementary file 1 — Additional file 1: Supplementary Table S1. Model inputs for DCC clinical efficacy, CHX treatment assumptions and sector care for patients contracting omphalitis. [file 12913_2021_6971_MOESM1_ESM.docx]

## Additional file 1: Supplementary Table 1. Model inputs for DCC clinical efficacy, CHX treatment assumptions and sector care for patients contracting omphalitis.

| **Variable** | **Base case** | **Lower*** | **Upper*** | **Reference** |
| --- | --- | --- | --- | --- |
|  |  |  |  |  |
| Omphalitis incidence with DCC (rate) | 0.170 | 0.136 | 0.203 | Cochrane review [[15](#_ENREF_15)]  (data supporting WHO guideline). Derived using weighted (inverse variance weight) from trials included in [[15](#_ENREF_15)]. Lower and upper based on +/- 20%. |
| CHX efficacy (relative risk of omphalitis vs DCC) | 0.730 | 0.584 | 0.876 | [[15](#_ENREF_15)] Lower and upper based on +/- 20%. |
| **Sector of care for patients contracting omphalitis** |  |  |  |  |
| Proportion of patients treated in public system | 0.60 | Calculated | Calculated | Calculated based on other inputs to sum to 1. |
| Proportion of patients treated in private system | 0.175 | 0.140 | 0.210 | Assumption. Lower and upper +/- 20% base case. |
| Proportion of patients treated in FBO | 0.175 | 0.140 | 0.210 |  |
| Proportion of patients untreated | 0.05 | 0.040 | 0.060 |  |

*Upper and lower values refer to corresponding values for each parameter in the sensitivity analysis.

CHX, chlorhexidine; DCC, dry cord care; FBO, faith-based organisation; WHO, World Health Organization.
