# Supplementary material for: Cost and consequences of using 7.1 % chlorhexidine gel for newborn umbilical cord care in Kenya
Source: BMC Health Serv Res. 2021 Nov 19;21:1249. doi: 10.1186/s12913-021-06971-7 (PMC8603569; doi:10.1186/s12913-021-06971-7)
Supplement: Supplementary file 2 — Additional file 2: Supplementary Table S2. Model inputs for hospitalisation (inpatient) and outpatient resource costs and medications costs (KSH). [file 12913_2021_6971_MOESM2_ESM.docx]

## Additional file 2: Supplementary Table 2. Model inputs for hospitalisation (inpatient) and outpatient resource costs and medications costs (KSH).

| **Variable** | **Data input** | **Lower*** | | **Upper*** | | **Reference** |  |
| --- | --- | --- | --- | --- | --- | --- | --- |
| **Proportion of patients with omphalitis treated in each setting** |  |  | |  | |  |  |
| Public system |  |  | |  | |  |  |
| Outpatient | 0.60 | Calculated | | Calculated | | Calculated as 1-outpatient proportion |  |
| Inpatient | 0.40 | 0.32 | | 0.48 | | Assumption. Lower and upper +/- 20% base case. |  |
| Private system |  |  | |  | |  |  |
| Outpatient | 0.60 | Calculated | | Calculated | | Calculated as 1-outpatient proportion |  |
| Inpatient | 0.40 | 0.32 | | 0.48 | | Assumption. Lower and upper +/- 20% base case. |  |
| FBO system |  |  | |  | |  |  |
| Outpatient | 0.60 | Calculated | | Calculated | | Calculated as 1-outpatient proportion |  |
| Inpatient | 0.40 | 0.32 | | 0.48 | | Assumption. Lower and upper +/- 20% base case. |  |
| **Proportion of patients treated in each hospital level** |  |  | |  | |  |  |
| Primary level hospital (public) | 0.50 | Calculated | | Calculated | | Calculated based on other inputs to sum to 1. |  |
| Secondary level hospital (public) | 0.40 | 0.32 | | 0.48 | | Assumption based on clinical opinion |  |
| Teaching hospital (public) | 0.10 | 0.08 | | 0.12 | | Assumption based on clinical opinion |  |
| Primary level hospital (private) | 0.50 | Calculated | | Calculated | | Calculated based on other inputs to sum to 1. |  |
| Secondary level hospital (private) | 0.40 | 0.32 | | 0.48 | | Assumption based on clinical opinion |  |
| Teaching hospital (private) | 0.10 | 0.08 | | 0.12 | | Assumption based on clinical opinion |  |
| Primary level hospital (FBO) | 0.50 | Calculated | | Calculated | | Calculated based on other inputs to sum to 1. |  |
| Secondary level hospital (FBO) | 0.40 | 0.32 | | 0.48 | | Assumption based on clinical opinion |  |
| Teaching hospital (FBO) | 0.10 | 0.08 | | 0.12 | | Assumption based on clinical opinion |  |
| **Length of inpatient stay (days)** |  |  | |  | |  |  |
| Public system | 5 | 4 | | 6 | | Clinical opinion |  |
| Private system | 5 | 4 | | 6 | | Clinical opinion |  |
| FBO system | 5 | 4 | | 6 | | Clinical opinion |  |
| **Hospitalisation cost per day (KSH)** |  |  | |  | |  |  |
| Public system |  |  | |  | |  |  |
| Primary level hospital | 813.90 | - | | - | | World Health Organization, 2008 [[25](#_ENREF_25)] |  |
| Secondary level hospital | 849.10 | - | | - | | World Health Organization, 2008 [[25](#_ENREF_25)] |  |
| Teaching hospital | 1,097.94 | - | | - | | World Health Organization, 2008 [[25](#_ENREF_25)] |  |
| Private system |  |  | |  | |  |  |
| Primary level hospital | 1,049.26 | - | | - | | World Health Organization, 2008 [[25](#_ENREF_25)] |  |
| Secondary level hospital | 1,094.68 | - | | - | | World Health Organization, 2008 [[25](#_ENREF_25)] |  |
| Teaching hospital | 1,415.46 | - | | - | | World Health Organization, 2008 [[25](#_ENREF_25)] |  |
| FBO system |  |  | |  | |  |  |
| Primary level hospital | 939.95 | - | | - | | World Health Organization, 2008 [[25](#_ENREF_25)] |  |
| Secondary level hospital | 980.59 | - | | - | | World Health Organization, 2008 [[25](#_ENREF_25)] |  |
| Teaching hospital | 1,267.89 | - | | - | | World Health Organization, 2008 [[25](#_ENREF_25)] |  |
| **Outpatient appointments** |  |  | |  | |  |  |
| Number of outpatient appointment visits per omphalitis infection |  |  | |  | |  |  |
| Public system | 1.0 | 0.8 | | 1.2 | | Clinical opinion |  |
| Private system | 1.0 | 0.8 | | 1.2 | | Clinical opinion |  |
| FBO system | 1.0 | 0.8 | | 1.2 | | Clinical opinion |  |
| **Outpatient cost per visit** |  |  | |  | |  |  |
| Public system |  |  | |  | |  |  |
| Health centre (no beds) (KSH) | 148.22** | - | | - | | World Health Organization, 2008 [[25](#_ENREF_25)] |  |
| Health centre (with beds) (KSH) | 182.99** | - | | - | | World Health Organization, 2008 [[25](#_ENREF_25)] |  |
| Primary level hospital (KSH) | 208.64** | - | | - | | World Health Organization, 2008 [[25](#_ENREF_25)] |  |
| Average primary level hospital | 179.95 | - | | - | | Calculated as average of health centre and primary level hospital based on clinical opinion |  |
| Secondary level hospital (KSH) | 217.33 | - | | - | | World Health Organization, 2008 [[25](#_ENREF_25)] |  |
| Teaching level hospital (KSH) | 217.33 | - | | - | | Assumption |  |
| Private system |  |  | |  | |  |  |
| Health centre (no beds) (KSH) | 209.07** | - | | - | | World Health Organization, 2008 [[25](#_ENREF_25)] |  |
| Health centre (with beds) (KSH) | 257.97** | - | | - | | World Health Organization, 2008 [[25](#_ENREF_25)] |  |
| Primary level hospital (KSH) | 293.83** | - | | - | | World Health Organization, 2008 [[25](#_ENREF_25)] |  |
| Average primary level hospital | 253.62 | - | | - | |  |  |
| Secondary level hospital (KSH) | 306.43 | - | | - | | World Health Organization, 2008 [[25](#_ENREF_25)] |  |
| Teaching level hospital (KSH) | 306.43 | - | | - | | Assumption |  |
| FBO system |  |  | |  | |  |  |
| Health centre (no beds) (KSH) | 198.20** | - | | - | | World Health Organization, 2008 [[25](#_ENREF_25)] |  |
| Health centre (with beds) (KSH) | 244.71** | - | | - | | World Health Organization, 2008 [[25](#_ENREF_25)] |  |
| Primary level hospital (KSH) | 278.83** | - | | - | | World Health Organization, 2008 [[25](#_ENREF_25)] |  |
| Average primary level hospital | 240.58 | - | | - | |  |  |
| Secondary level hospital (KSH) | 290.57 | - | | - | | World Health Organization, 2008 [[25](#_ENREF_25)] |  |
| Teaching level hospital (KSH) | 290.57 | - | | - | | Assumption |  |
| **Medication costs** |  | |  | |  |  | |
| Gentamicin |  |  |  |  |  |  |  |
| Cost of per pack (KSH) | 200.00 | | 160.00 | | 240.00 | KEMSA, 2019 [[33](#_ENREF_33)] | |
| Dose (day) (mg/kg) | 5 | | - | | - | Kenya Ministry of Health, 2016 [[27](#_ENREF_27)] | |
| Treatment length (days) | 5 | | - | | - | Clinical Opinion | |
| mg per vial | 20 | | - | | - | KEMSA, 2019 [[33](#_ENREF_33)] | |
| Number of vials in pack | 25 | | - | | - | KEMSA, 2019 [[33](#_ENREF_33)] | |
| Penicillin (benzyl penicillin) |  |  |  |  |  |  |  |
| Cost of injection (KSH) | 28.00 | | 22.40 | | 33.60 | KEMSA, 2019 [[33](#_ENREF_33)] | |
| Dose (day) (IU/kg) | 100,000 | | - | | - | Kenya Ministry of Health, 2016 [[27](#_ENREF_27)] | |
| Treatment length (days) | 5 | | - | | - | Clinical Opinion | |
| IU per vial | 5,000,000 | | - | | - | KEMSA, 2019 [[33](#_ENREF_33)] | |
| Oral penicillin (amoxicillin) |  |  |  |  |  |  |  |
| Cost per pack (KSH) | 360.00 | | 288.00 | | 432.00 | KEMSA, 2019 [[33](#_ENREF_33)] | |
| Tablets per pack | 10 | | - | | - | KEMSA, 2019 [[33](#_ENREF_33)] | |
| Dose (day) (mg/kg) | 50 | | - | | - | Kenya Ministry of Health, 2016 [[27](#_ENREF_27)] | |
| Treatment length (days) | 5 | | - | | - | Clinical Opinion | |
| mg per tablet | 228.5 | | - | | - | KEMSA, 2019 [[33](#_ENREF_33)] | |
| Paracetamol (oral suspension) |  |  |  |  |  |  |  |
| Cost per treatment (KSH) | 30.00 | | 24.00 | | 36.00 | KEMSA, 2019 [[33](#_ENREF_33)] | |
| Dose (day) (mg/kg) | 15 | | - | | - | Clinical Opinion | |
| Treatment length (days) | 7 | | - | | - | Clinical Opinion | |
| Amount in vial (mg) | 120 | | - | | - | KEMSA, 2019 [[33](#_ENREF_33)] | |
| Amount per vial (mL) | 100 | | - | | - | KEMSA, 2019 [[33](#_ENREF_33)] | |
| *Upper and lower values refer to corresponding values for each parameter in the sensitivity analysis.  **To reflect the structure of Kenya’s outpatient facilities, the WHO CHOICE categorical breakdown (Health centre [no beds], health centre [with beds], primary level hospital) were averaged to provide an input used to populate the cost for primary care appointments labelled “Average Primary Level Hospital” in the model.  FBO, faith-based organisation; IU, international units; KSH, Kenyan shilling; WHO, World Health Organization. | | | | | | | |

**Reference**

33. KEMSA LMIS Price List 2019 [http://www.kemsa.co.ke/wp-content/uploads/2019/06/KEMSA_LMIS-price-list-June-2019.xls]
