# Supplementary material for: Cost and consequences of using 7.1 % chlorhexidine gel for newborn umbilical cord care in Kenya
Source: BMC Health Serv Res. 2021 Nov 19;21:1249. doi: 10.1186/s12913-021-06971-7 (PMC8603569; doi:10.1186/s12913-021-06971-7)
Supplement: Supplementary file 3 — Additional file 3: Supplementary Table S3. Model inputs for proportion of patients treated with each drug regimen. [file 12913_2021_6971_MOESM3_ESM.docx]

## Additional file 3: Supplementary Table 3. Model inputs for proportion of patients treated with each drug regimen.

|  | **Public** | | **Private** | | **FBO** | | **Reference** |
| --- | --- | --- | --- | --- | --- | --- | --- |
|  | **Inpatient** | **Outpatient** | **Inpatient** | **Outpatient** | **Inpatient** | **Outpatient** | **Inpatient** |
| Gentamicin and penicillin (injectable) | 1 | 0 | 1 | 0 | 1 | 0 | Clinical opinion |
| Oral penicillin (amoxicillin) | 1 | 1 | 1 | 1 | 1 | 1 | Clinical opinion |
| Paracetamol (oral suspension) | 1 | 1 | 1 | 1 | 1 | 1 | Clinical opinion |
| Other medication (user defined) | 0 | 0 | 0 | 0 | 0 | 0 | Clinical opinion |

FBO, faith-based organisation.
