# Supplementary material for: Cost and consequences of using 7.1 % chlorhexidine gel for newborn umbilical cord care in Kenya
Source: BMC Health Serv Res. 2021 Nov 19;21:1249. doi: 10.1186/s12913-021-06971-7 (PMC8603569; doi:10.1186/s12913-021-06971-7)
Supplement: Supplementary file 4 — Additional file 4: Supplementary Table S4. Model inputs for non-medication costs. [file 12913_2021_6971_MOESM4_ESM.docx]

## Additional file 4: Supplementary Table 4. Model inputs for non-medication costs.

| **Variable** | **Cost input**  **(KSH)** | **Lower***  **(KSH)** | **Upper***  **(KSH)** | **Reference** |
| --- | --- | --- | --- | --- |
| **Consumable non-medication items costs (all sectors)** |  |  |  |  |
| Gloves cost per pair | 5.90 | 4.70 | 7.10 | Clinical opinion |
| Syringe cost | 5.30 | 4.20 | 6.40 | Clinical opinion |
| Saline solution cost per 500 mL | 40.00 | 32.0 | 48.0 | Clinical opinion |
| **Laboratory test costs by healthcare sector** |  |  |  |  |
| Public system |  |  |  |  |
| Haemogram | 200.00 | 160.00 | 240.00 | Clinical Opinion |
| Blood culture | 410.00 | 328.00 | 492.00 | Clinical Opinion |
| Umbilical cord swab | 410.00 | 328.00 | 492.00 | Clinical Opinion |
| Private system |  |  |  |  |
| Haemogram | 1299.00 | 1039.20 | 1558.80 | Pathologist Lancet Kenya Price List, 2017[[28](#_ENREF_28)] |
| Blood culture | 6199.00 | 4959.20 | 7438.80 | Pathologist Lancet Kenya Price List, 2017[[28](#_ENREF_28)] |
| Umbilical cord swab | 99.00 | 79.20 | 118.80 | Pathologist Lancet Kenya Price List, 2017[[28](#_ENREF_28)] |
| FBO system |  |  |  |  |
| Haemogram | 433.00 | 346.40 | 519.60 | Clinical opinion |
| Blood culture | 2066.33 | 1653.10 | 2479.60 | Clinical Opinion |
| Umbilical cord swab | 33.00 | 26.40 | 39.60 | Clinical Opinion |
| *Upper and lower values refer to corresponding values for each parameter in the sensitivity analysis.  FBO, faith-based organisation; KSH, Kenyan shilling. | | | | |
