# Supplementary material for: Cost and consequences of using 7.1 % chlorhexidine gel for newborn umbilical cord care in Kenya
Source: BMC Health Serv Res. 2021 Nov 19;21:1249. doi: 10.1186/s12913-021-06971-7 (PMC8603569; doi:10.1186/s12913-021-06971-7)
Supplement: Supplementary file 5 — Additional file 5: Supplementary Table S5. Model inputs for number of laboratory tests and consumable non-medication items conducted per case of omphalitis. [file 12913_2021_6971_MOESM5_ESM.docx]

## Additional file 5: Supplementary Table 5. Model inputs for number of laboratory tests and consumable non-medication items conducted per case of omphalitis.

|  | **Public** | | **Private** | | **FBO** | | **Reference** |
| --- | --- | --- | --- | --- | --- | --- | --- |
|  | **Inpatient** | **Outpatient** | **Inpatient** | **Outpatient** | **Inpatient** | **Outpatient** | **Inpatient** |
| Haemogram | 1 | 1 | 1 | 1 | 1 | 1 | Clinical Opinion |
| Blood culture | 0 | 0 | 0 | 0 | 0 | 0 | Clinical Opinion |
| Umbilical cord swab | 0 | 0 | 1 | 1 | 1 | 1 | Clinical Opinion |
| Gloves | 15 | 1 | 15 | 1 | 15 | 1 | Clinical Opinion |
| Syringes | 15 | 0 | 15 | 0 | 15 | 0 | Clinical Opinion |
| Saline solution | 1 | 0 | 1 | 0 | 1 | 0 | Clinical Opinion |

FBO, faith-based organisation.
