# Supplementary material for: Cost and consequences of using 7.1 % chlorhexidine gel for newborn umbilical cord care in Kenya
Source: BMC Health Serv Res. 2021 Nov 19;21:1249. doi: 10.1186/s12913-021-06971-7 (PMC8603569; doi:10.1186/s12913-021-06971-7)
Supplement: Supplementary file 6 — Additional file 6: Supplementary Table S6: Model inputs for indirect costs. [file 12913_2021_6971_MOESM6_ESM.docx]

## Additional file 6: Supplementary Table 6. Model inputs for indirect costs.

| **Variable** | **Data Input** | **Lower*** | **Upper*** | **Reference** |
| --- | --- | --- | --- | --- |
| Average gross salary (KSH) | 684,097.00 | 547,278 | 820,916 | Kenya National Bureau of Statistics, 2018[[34](#_ENREF_34)] |
| Caregiver days off | 5 | 1 | 6 | Assumption (based on expert opinion) |

*Upper and lower values refer to corresponding values for each parameter in the sensitivity analysis.

KSH, Kenyan shilling.

**Reference**

34. Economic Survey 2018 [https://www.knbs.or.ke/download/economic-survey-2018/]
