# Supplementary material for: Cost and consequences of using 7.1 % chlorhexidine gel for newborn umbilical cord care in Kenya
Source: BMC Health Serv Res. 2021 Nov 19;21:1249. doi: 10.1186/s12913-021-06971-7 (PMC8603569; doi:10.1186/s12913-021-06971-7)
Supplement: Supplementary file 7 — Additional file 7: Supplementary Table S7: Rationale for the included literature in this cost-consequence analysis. [file 12913_2021_6971_MOESM7_ESM.docx]

Additional file 7: Supplementary Table 7. Rationale for the included literature in this cost-consequence analysis.

| **Literature source** | **Rationale for hierarchy** | **Limitations** | **Appropriateness for populating Kenyan model** |
| --- | --- | --- | --- |
| 1. Imdad et al. (2013) [[24](#_ENREF_24)] | Cochrane systematic literature review and meta-analysis – well-conducted and appropriate meta-analyses are viewed as the pinnacle of the hierarchy of evidence [[35](#_ENREF_35)]. Therefore, this meta-analysis, which provides the largest pool of data, synthesising evidence from 3 RCTs, is perceived to be the most robust data source in supporting the efficacy assumption for the cost-consequence model for CHX. The evidence synthesis undertaken provided an increase in precision in relative effectiveness due to an increase in sample size. The analysis conducted was of high quality and has been accepted by the community as providing an indication of the effectiveness of CHX versus DCC. This source was used to support the WHO recommendation of CHX for cord care. | Limitations of the generalisability to Kenya of this source include the studies being undertaken in Asia where at-home birth rates were higher, potentially increasing the risk of infection relative to Kenya. | High. Most suitable for use as the base case due to the strength of evidence attributable to the meta-analysis and acceptance by WHO. |
| Sazawal et al. (2016)[[16](#_ENREF_16)] | This RCT was undertaken on Pemba Island, Tanzania, which geographically borders Kenya. At-home birth rates were somewhat similar to that observed in Kenya, which supports the clinical opinion that there are similarities in the provision of obstetric and postnatal care between countries. | The paper noted within the discussion, the level of awareness of appropriate DCC on Pemba Island at the time of this study was high, and may not therefore be reflective of the general quality of DCC when used in usual practice. This may have contributed to the relatively low neonatal mortality rate of 11.7 per 1000 observed in the DCC arm which led to challenges in interpreting the primary outcome. | Medium. Suitable for use as a sensitivity analysis due to healthcare system similarities, including settings of birth. |
| Semrau (2016)[[17](#_ENREF_17)] | This RCT (ZamCAT) was undertaken in Zambia, with comparable home-birth rates to Kenya. | The data from this trial should be interpreted and applied with consideration: although the ZamCAT study was large (~18–19000 participants in each arm), there were several limitations to the study, highlighted by the authors in the manuscript. The key limitations were as follows: “(T)he observed neonatal mortality rate in the DCC group was 50% lower than the expected neonatal mortality rate (37 deaths per 1000 livebirths).” This lower-than-expected rate of neonatal mortality rate may be due to the package of services, that all women, regardless of intervention, received and therefore this may have resulted in a better standard of cord care practices in the trial than in clinical practice. Incidence rates of omphalitis in both arms and use of traditional medicines on the cord were also far lower than expected by authors, lower than experts believe to be occurring in clinical practice, and far lower than reported by other studies available, potentially reinforcing this point. The authors also note that it is “possible that mild or moderate cases were under-reported as diagnosis was based on purulent discharge or redness at the umbilical stump.” | Low. To be considered as an exploratory analysis only predominantly due to the lack of alignment in incidence rate of omphalitis and DCC practices in this study compared to Kenya (based on clinical opinion). |

CHX, chlorhexidine; DCC, dry cord care; RCT, randomised controlled trial; WHO, World Health Organization.

**Reference**

35. Burns PB, Rohrich RJ, Chung KC: The levels of evidence and their role in evidence-based medicine. Plast Reconstr Surg 2011, 128(1):305–310.
